# Effects of fish oil supplementation on bone turnover markers in depression: a pilot study

**DOI:** 10.3389/fnut.2024.1464526

**Published:** 2024-12-12

**Authors:** Feifei Wang, Hui Yuan, Kun Jin, Hui Tang, Jimin Guo, Chuan-Yue Wang, Jindong Chen, Fang Dong, Lu Wang

**Affiliations:** ^1^The National Clinical Research Center for Mental Disorders and Beijing Key Laboratory of Mental Disorders and Beijing Institute for Brain Disorders Center of Schizophrenia, Beijing Anding Hospital, Capital Medical University, Beijing, China; ^2^Advanced Innovation Center for Human Brain Protection, Capital Medical University, Beijing, China; ^3^Department of Stomatology, The Second Xiangya Hospital, Central South University, Changsha, China; ^4^Department of Psychiatry, National Clinical Research Center for Mental Disorders, National Center for Mental Disorders, and China National Technology Institute on Mental Disorders, The Second Xiangya Hospital of Central South University, Changsha, Hunan, China; ^5^College of Materials Sciences and Engineering, Beijing University of Chemical Technology, Beijing, China

**Keywords:** major depressive disorder, n-3 PUFA, bone formation, bone resorption, bone loss

## Abstract

**Background and objective:**

There is a close correlation between bone loss, depression, and antidepressants. N-3 PUFA supplementation has been considered an effective add-on therapeutic approach in ameliorating bone loss and relieving depression. However, the adjunctive effect of n-3 PUFA on bone metabolism in participants with depression is still unknown. This is a pilot study to investigate the dynamics of bone metabolism in depression and evaluate the efficacy of fish oil on bone loss in depression.

**Methods:**

In this study, we focused on the change of bone turnover markers in depression, the effect of n-3 PUFA supplementation on bone turnover markers, and its association with clinical characteristics. A case–control study and a secondary analysis of a previously published randomized clinical trial (NCT03295708) that evaluates the efficacy of n-3 PUFA supplementation in venlafaxine-treated depressed participants have been included.

**Results:**

The levels of PINP (z = −2.233, *p* = 0.026) in depressed participants were significantly increased compared with healthy controls at baseline. The secondary analysis has shown significant differences exited on CTX (*χ*^2^ = 4.848, *p* = 0.028) and OSTEOC (*χ*^2^ = 6.178, *p* = 0.013) between n-3 PUFA and placebo group. The levels of CTX and OSTEOC (*p* < 0.05) significantly decreased in the placebo group, which indicates that venlafaxine treatment reduces both bone formation and resorption markers. While the levels of OSTEOC and PINP were increased in the n-3 PUFA group (*p* < 0.05). Moreover, the change in bone turnover markers showed consistency with clinical symptomatic outcomes.

**Conclusion:**

Participants with first-diagnosed, drug-naïve depression show active bone formation. Venlafaxine decreases bone remodeling, while n-3 PUFA increases bone formation, bringing light to preventing and treating bone loss in depression.

**Clinical trial registration:**

ClinicalTrials.gov, NCT03295708.

## 1 Introduction

Major depressive disorder (MDD) increases the prevalence of bone loss, osteoporosis, and fractures, which belong to chronic systemic skeletal disorders caused by an imbalance between bone formation and bone resorption (1–4). Osteoporosis has also been proven to be an independent risk factor for depression (5). These bone metabolism-related comorbidities induce substantial morbidity and mortality (6). In addition to changes in lifestyle and behaviors (2), changes in the endocrine and immune systems secondary to depression also play an essential role in bone metabolism (7). Not only depression itself, but the use of antidepressants also increases the risk of bone loss and fractures (8, 9). Selective serotonin reuptake inhibitors (SSRIs) and serotonin and norepinephrine reuptake inhibitors (SNRIs), being the first-line pharmaceutical treatment, have been widely prescribed in recent years (10). In addition, fluvoxamine was found to accumulate in bone marrow at concentrations much higher than those in the peripheral blood, suggesting that antidepressants can bioaccumulate in bone (11). The potential effect of antidepressants on bone metabolism deserves attention.

The molecular mechanisms underlying the effects of antidepressants on bone metabolism are not fully understood. SSRIs users have twice the rate of bone loss in the hip and a higher risk of osteoporosis and related fractures compared with non-users (12). This might be because peripheral 5-HT could act on osteoblasts and inhibit bone formation (13, 14). Furthermore, the risks of fractures between SNRIs and SSRIs users were comparable (15). Apart from 5-HT, SNRIs also can inhibit NE (norepinephrine) reuptake. The inhibition of NE reuptake affects the sympathetic nervous system, strongly influencing osteoclast and osteoblast function (16, 17). Additionally, after long-term pharmacological NE transport blockade, the genetic ablation of NE transporter, expressed in bone cells, leads to a low peak bone mass (18, 19).

Since treatment recommendations for osteoporosis are often based on future fracture risk, and some anti-osteoporosis drugs have serious adverse effects (20), potential options for bone loss from a preventive perspective are of great importance. Accumulating evidence has established the essential roles of PUFA in bone metabolism (21). N-3 PUFA, mainly consisting of eicosapentaenoic acid (EPA) and docosahexaenoic acid (DHA), may positively impact bone remodeling by reducing osteoclast activity and increasing osteoblast activity (22, 23). They could also facilitate bone metabolism through prostaglandin production, calcium absorption, and lipid oxidation (24–26). Depressed participants will develop bone metabolism-related problems in the early stages of the disease, and long-term use of antidepressants is likely to increase bone loss and related risks. As a result, it is essential to evaluate bone metabolism activity in the early stages of depression and find possible preventive solutions.

This study aimed to investigate the dynamics of bone metabolism in depression, the efficacy of n-3 PUFA supplementation on bone metabolism, and finally, the association between bone metabolism and clinical characteristics. By investigating these critical aspects, the research would offer significant perspectives that may eventually result in enhanced interventions and tactics targeted at improving disease prognosis and ultimately improving the quality of life in depression.

## 2 Methods

### 2.1 Study design

**Figure 1:**
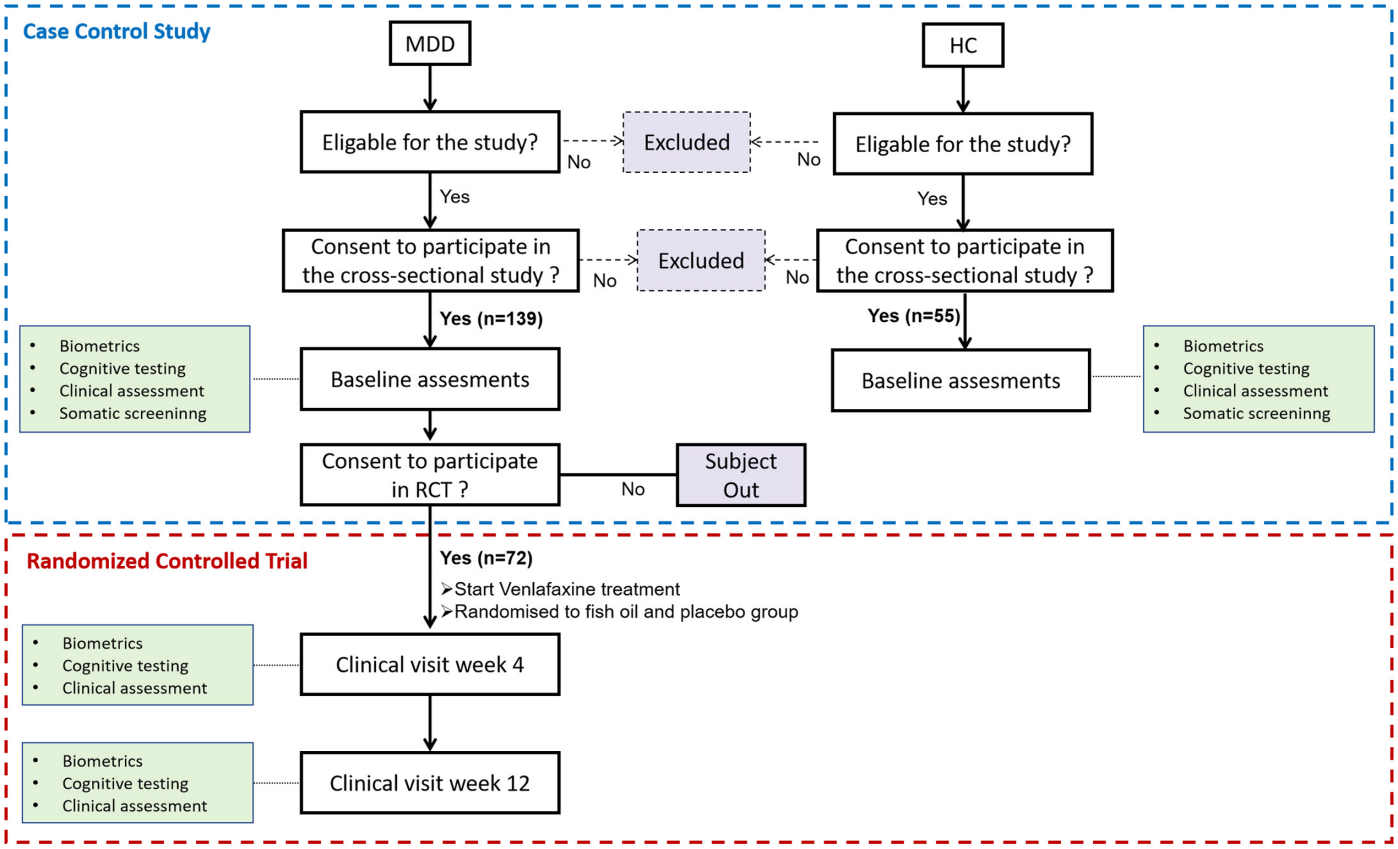
Flowchart of study trial assessments for participants.

This study’s clinical data and biomarkers came from a case–control study and a randomized, double-blind, placebo-controlled trial (RCT). Figure 1 shows a flowchart of the scheduled data collection of the case–control study and RCT over the 12 weeks of treatment of participants with MDD. The cross-sectional study included 139 first-diagnosed, drug-naïve participants with depression and 55 healthy controls (HCs) as previously published (27). The randomized, double-masked, placebo-controlled trial recruited venlafaxine-treated depressed participants had two groups (n = 36 per group): 2.4 g/d n-3 PUFA (1,440 mg of eicosapentaenoic acid [EPA] plus 960 mg of docosahexaenoic acid [DHA]); and placebo (soybean oil) groups. The detailed protocol and primary outcomes, which mainly evaluate the effectiveness and safety, were published (28).

### 2.2 Study participants

All participants provided written informed consent. Participants were 18- to 50-year-old outpatients or HCs recruited from the Second Xiangya Hospital of Central South University from March 2017 to January 2020. Two specialists in psychiatry evaluated participants, and all participants met the Diagnostic and Statistical Manual of Mental Disorders IV criteria for MDD. The following were the inclusion criteria: (1) Hamilton Depression Rating Scale (24-item HAMD) score greater than 20 points; (2) first-time diagnosis and non-use of antipsychotics. Exclusion criteria included: (1) a history or current of a clinically significant disease; (2) pregnancy or breastfeeding; (3) Current apparent suicide attempts or suicidal behavior; (4) any conditions or drugs that may affect biomarkers: long-term regular use of NSAIDs, COX-2 inhibitors, immunosuppressants, hormone, interferons, chemotherapy drugs, anticoagulants; (5) taking supplements containing omega-3 PUFAs or eating omega-3-rich fatty fish more than twice a week. A total of 139 subjects met the inclusion criteria, and ultimately, 72 participated in the RCT evaluating the efficacy of n-3 PUFA in the adjunctive treatment of depression. A previous study described HCs as healthy volunteers with no psychiatric history or medical conditions (27). The clinical trial was approved by the Ethics Committee of the Second Xiangya Hospital of Central South University (MDD201610).

### 2.3 Clinical assessment

Demographic and clinical information, including gender, age, and BMI, were collected from each subject at baseline. Assessments of the 24-item Hamilton Depression Rating Scale (HAMD-24), Hamilton Anxiety Rating Scale (HAMA), Zung Self-Rating Anxiety Scale (SAS), and Beck Depression Inventory (BDI) were completed at baseline, week 4, and week 12 of treatment.

### 2.4 Sample processing and bone turnover markers assessment

Between 6 and 9 a.m., the participants’ blood was gathered while fasting at baseline, clinical visit week 4, and clinical visit week 12. Bone turnover markers were assessed on baseline and clinical visit 12. The blood samples were centrifuged at 3,000 rpm for 10 min, and the plasma was removed and stored in a refrigerator at −80°C. A Roche Cobas 8000-e602 automated electrochemiluminescence immunoassay system was used to measure the levels of C-terminal crosslinked terminal peptide of type I collagen (CTX), thyrocalcitonin (hCT), osteocalcin (OSTEOC), parathyroid hormone (PTH), and N-terminal propeptides of type I collagen (PINP). The assessment was conducted at the Second Xiangya Hospital of Central South University. The study assistants were blinded to the grouping of samples during any evaluation process.

### 2.5 Statistical analysis

Data analyses were performed using IBM SPSS Statistics 26.0 and GraphPad Prism 8.0. Baseline data that conformed to the normal distribution were analyzed using independent samples t-tests. Data were presented in mean (SD), and chi-square tests or Fisher’s exact probability method were used for statistical analysis of count data. Non-normal data were expressed as median (P25-P75), and repeated measures were analyzed using a generalized estimating equation (GEE). Linear regression analyses examined associations between bone turnover markers (baseline levels and post-intervention changes) and clinical outcomes. All statistical significance tests were performed using two-tailed tests, and *p* < 0.05 was considered a statistically significant difference.

## 3 Results

### 3.1 Demographic and clinical characteristics

A comparison of demographic data and clinical characteristics between the MDD and HC groups is shown in Table 1. There were no significant differences in age, gender, and BMI between the MDD and HC groups. In addition, depression and anxiety self-rating scale analysis showed that the BDI (*p* < 0.001) and SAS (*p* < 0.001) scores of depression participants were significantly higher than those of healthy controls.

**Table 1:** Baseline characteristics of HC and MDD groups.

Parameter	HCs (*n* = 55)	MDD (*n* = 139)	t/*χ*^2^	*p* value
Age	29.1(8.8)	27.6(8.7)	−1.100	0.273
Female, *n* (%)	31(56.4)	91(65.5)	1.399	0.237
BMI (kg/m^2^)	21.9(2.7)	21.4(2.9)	−1.121	0.264
HAMD	-	29.7(6.7)	-	-
HAMA	-	22.7(6.7)	-	-
BDI	5.5(5.3)	28.5(10.0)	20.127	<0.001**
SAS	39.7(7.7)	55.5(11.2)	10.870	<0.001**

BMI, body mass index; BDI, Beck Depression Inventory; HAMA, Hamilton Anxiety Scale; HAMD, 24-item Hamilton Depression Rating Scale; HC, healthy control; MDD, major depressive disorder; SAS, Self-Rating Anxiety Scales. **p < 0.01. The bold values indicate the data with statistical significance.

### 3.2 Changes in bone turnover markers between HCs and participants with depression

Table 2 lists the results of bone turnover markers and differences between the HC and MDD groups. The levels of PINP (z = −2.233, *p* = 0.026) in the depression participants were significantly increased compared with the HCs. In contrast, the CTX, hCT, OC, and PTH levels were not significantly different between the two groups.

**Table 2:** Bone turnover markers between HC and MDD groups.

	HC (*n* = 55)	MDD (*n* = 139)	Z	*p* value
CTX	0.37 (0.24–0.47)	0.38 (0.28–0.53)	−0.734	0.463
hCT	1.38 (0.50–2.26)	1.25 (0.50–2.31)	−0.352	0.725
OSTEOC	17.60 (13.91–21.30)	19.74 (15.03–23.53)	−0.446	0.656
PTH	33.83 (27.37–43.03)	34.09 (26.20–44.14)	−0.175	0.861
PINP	56.85 (43.13–70.24)	64.19 (48.78–83.74)	−2.233	**0.026** ^*^

The bone turnover markers were assessed at baseline. CTX: C-terminal crosslinked terminal peptide of type I collagen; hCT: thyrocalcitonin; OC: osteocalcin; PTH: parathyroid hormone; PINP: N-terminal propeptides of type I collagen. Data are presented as interquartile range (P25–P75). *p < 0.05. The bold values indicate the data with statistical significance.

### 3.3 Bone turnover marker change after 12-week treatment

**Figure 2:**
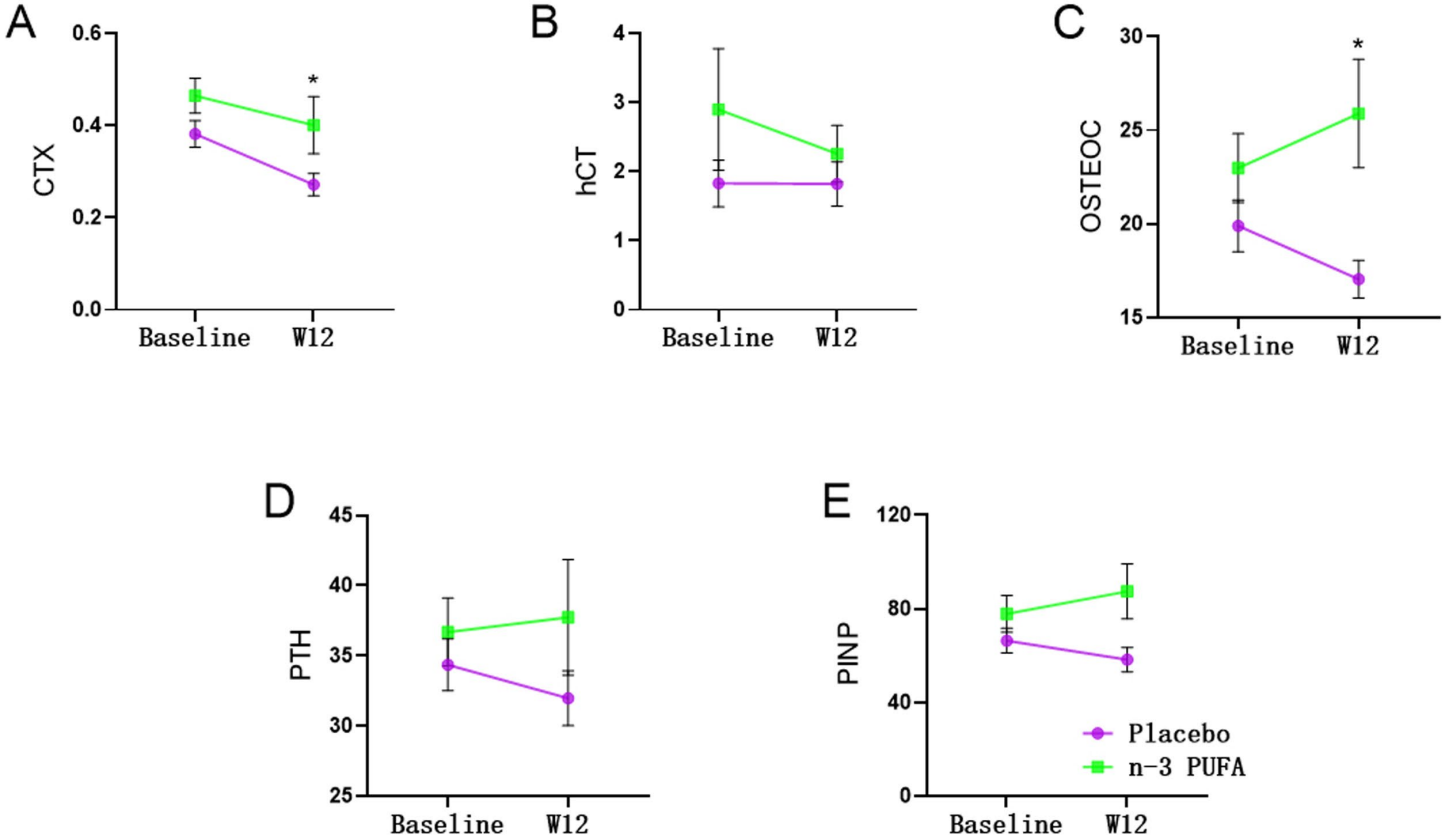
Changes of bone turnover markers between placebo and n-3 PUFA groups *: significantly different after n-3 PUFA supplementation.

As shown in Table 3 and Figure 2, significant differences exited on CTX (*χ*^2^ = 4.848, *p* = 0.028) and OSTEOC (*χ*^2^ = 6.178, *p* = 0.013) between n-3 PUFA and placebo group. As for the PINP level (*χ*^2^ = 3.759, *p* = 0.053), there is a trend without statistical significance. After 12 weeks of treatment, both OSTEOC and PINP levels increased significantly in the n-3 PUFA group comparing the placebo group (*p* < 0.05).

**Table 3:** The bone turnover markers between the two groups at baseline and 12 weeks after treatment.

	Baseline		W12		Intervention *χ*^2^ (*p*-value)	Time *χ*^2^ (*p*-value)	Intervention + time *χ*^2^ (*p*-value)
	Placebo	n-3 PUFA	Placebo	n-3 PUFA			
CTX	0.38 (0.03)	0.47 (0.04)	0.27 (0.02) ^**a**^	0.40 (0.06)	**4.848 (0.028)**	**7.188 (0.007)**	0.495 (0.482)
hCT	1.83 (0.34)	2.90 (0.88)	1.82 (0.32)	2.26 (0.41)	1.277 (0.258)	0.785 (0.376)	0.722 (0.396)
OSTEOC	19.89 (1.38)	22.98 (1.84)	17.04 (1.01) ^**a**^	25.90 (2.89) ^*^	**6.178 (0.013)**	0.001 (0.980)	**5.329 (0.021)**
PTH	34.36 (1.85)	36.69 (2.42)	31.98 (1.95)	37.74 (4.11)	1.944 (0.163)	0.068 (0.794)	0.447 (0.504)
PINP	66.33 (5.28)	77.87 (7.86)	58.24 (5.22)	87.38 (11.77) ^*^	3.759 (0.053)	0.028 (0.867)	**4.364 (0.037)**

CTX: C-terminal crosslinked terminal peptide of type I collagen; hCT: thyrocalcitonin; OSTEOC: osteocalcin; PTH: parathyroid hormone; PINP: N-terminal propeptides of type I collagen. Data are presented as mean ± SEM. *p < 0.05: significantly different between n-3 PUFAs and placebo group at different visit. ^a^*p* < 0.05: significantly different between baseline and week 12 in the n-3 PUFAs or placebo group. The bold values indicate the data with statistical significance.

After 12 weeks of treatment, CTX (*χ*^2^ = 7.188, *p* = 0.007) levels were significantly altered. In the placebo group, CTX level significantly decreased after 12 weeks of venlafaxine treatment (*p* < 0.05). In addition, the OSTEOC level decreased significantly after 12 weeks of venlafaxine treatment in the placebo group.

### 3.4 Associations between change in bone turnover markers and week 12 clinical outcome measures

After adjusting for baseline bone turnover marker levels and baseline score of clinical measure, linear regression analyses examining associations between bone turnover markers (baseline levels and post-intervention changes) with clinical outcomes revealed that a decrease in CTX level during the intervention was significantly associated with more favorable outcomes on the SAS (Stand. Beta = 0.368, *p* = 0.030) and BDI (Stand. Beta = 0.452, *p* = 0.014), and an increase in hCT level was significantly associated with more favorable outcomes on the SAS (Stand. Beta = −0.845, *p* = 0.014). Furthermore, an increase in PINP level was significantly associated with more favorable outcomes on the HAMA (Stand. Beta = −0.321, *p* = 0.041) at week 12. A decreased CTX level predicted better symptomatic outcomes by self-rating scales. In addition, an increase in hCT level predicted better anxiety self-rating outcomes, and an increase in PINP level predicted better clinician-rating anxiety outcomes (Table 4).

**Table 4:** Associations between change in bone turnover markers and outcome measures at week 12.

Variables	Stand. Beta	95% CI	*p* value
Change CTX^a^			
HAMD	0.254	−4.433–22.617	0.181
HAMA	0.082	−8.422–13.185	0.657
BDI	0.452	5.137–43.167	**0.014**
SAS	0.368	1.470–27.317	**0.030**
Change hCT^a^			
HAMD	0.106	−2.285–2.865	0.820
HAMA	−0.046	−2.007–1.803	0.914
BDI	−0.445	−5.207–1.707	0.311
SAS	−0.845	−4.842–−0.212	**0.014**
Change OSTEOC^a^			
HAMD	0.000	−0.503–0.503	0.999
HAMA	−0.050	−0.452–0.334	0.763
BDI	0.156	−0.331–0.974	0.352
SAS	0.250	−0.064–0.866	0.089
Change PTH^a^			
HAMD	0.076	−0.145–0.219	0.685
HAMA	0.035	−0.123–0.150	0.844
BDI	0.199	−0.108–0.374	0.271
SAS	0.176	−0.080–0.265	0.287
Change PINP^a^			
HAMD	−0.266	−0.254–0.034	0.130
HAMA	−0.321	−0.204–−0.004	**0.041**
BDI	0.178	−0.085–0.289	0.278
SAS	0.095	−0.093–0.177	0.530

CTX: C-terminal crosslinked terminal peptide of type I collagen; hCT: thyrocalcitonin; OC: osteocalcin; PTH: parathyroid hormone; PINP: N-terminal propeptides of type I collagen. ^a^Change pre-and post-intervention. ^b^Based on linear regression analysis adjusted for baseline bone turnover marker levels and baseline score of clinical measure. The bold values indicate the data with statistical significance.

## 4 Discussion

This study investigated the changes in bone turnover markers in first-diagnosed, drug-naïve depression, and the efficacy of fish oil supplementation on bone turnover markers and its association with clinical outcomes were also investigated. The significant findings are as follows: (1) Bone formation maker (PINP) is increased in drug-free depressed participants. (2) 12 weeks of venlafaxine treatment decreases both bone formation (OSTEOC) and resorption (CTX) markers, and n-3 PUFA supplementation could increase bone formation markers (OSTEOC and PINP). (3) The decrease in bone resorption marker (CTX) and increase in bone formation marker (hCT and PINP) predicts better outcomes for anxiety symptoms.

Previous studies have shown that the severity of depressive symptom severity and early life stress in depression were positively associated with levels of CTX and PINP, which suggests that anabolic activation of bone metabolism occurs during a depressive episode (29, 30). In addition, the level of CTX was significantly increased in mice exposed to early-life stress (30). And chronic mild stress induces bone loss (31). Wippert et al. concluded that long-term decline in bone mineral density (BMD) in depressed participants may be linked to either an overshooting or a lack of metabolic adaptation during a depressive episode (29). Our previous study has also found that individuals with mood disorders (32) have lower BMD than healthy controls. However, the current research only observed elevated bone formation markers (PINP) and did not find alterations in bone resorption markers. This may be because depressed individuals already show low BMD and bone formation is compensatory enhanced. Meanwhile, the bone resorption process is either slowed or is not reflected by current markers. Furthermore, newly diagnosed, drug-naïve participants with bipolar disorder also showed increased compensatory bone formation, with increased levels of PINP, OSTEOC, and PTH (33), which is consistent with our findings. The exact mechanisms and potential confounders relating to the correlation between bone metabolism and depression remain to be elucidated.

Despite the effect of the disease itself on bone metabolism, antidepressants also have a clinically significant impact on bone metabolism (34), although with inconsistent findings. Previous studies have found that CTX increased and P1NP decreased during venlafaxine treatment in older adults with depression (35, 36). Our study found that venlafaxine treatment induces reduced bone metabolic activity (CTX and OSTEOC) among relatively younger depressed participants (mean age: 27.6 years). In addition, other studies have found that PTH and CTX decreased, and OSTEOC increased with 12 weeks of escitalopram treatment in premenopausal female participants with newly diagnosed depression (37), while 8 weeks of escitalopram treatment does not significantly alter bone turnover markers in peri- or postmenopausal non-depressed women (38). It seems that the type of antidepressant, how long it is used, and who uses it might influence the outcomes of bone turnover markers. Depressed individuals might be more susceptible to antidepressant-induced changes in bone metabolism.

Studies on animals have also demonstrated the complexity of SSRI’s effects on bone metabolism. Fluoxetine treatment significantly reduced osteocalcin levels in mice but with no significant impact on CTX, and desipramine (SNRIs) had no significant effect on either (39). What’s more, Maria et al. found that short-term use of fluoxetine (3 weeks) increased bone mass by directly impairing osteoclast differentiation and function, leading to an increase in local antiresorptive effects, whereas long-term (6 weeks) use of fluoxetine led to a decrease in bone mass because fluoxetine acted on adrenergic receptors on osteoblasts by increasing central sympathetic activity, thus decreasing bone formation (8). The differential effects of short- or long-term fluoxetine use on bone metabolism have been confirmed in other studies (40, 41). Since 5-HT-related receptors are also expressed in the skeletal system, this may be one of the reasons for the complex effects of SSRIs on bone. *In vitro* experiments have found that 5-HT or fluoxetine promotes the differentiation of human monocytes into osteoblast-like cells and increases their bone resorption (42). In contrast, high concentrations of fluoxetine have an inhibitory effect on osteoblast differentiation and activity (42, 43). Results from *in vivo* experiments have also been inconsistent: some studies have found that inhibition of 5-HT and 5-HT transporters has a protective effect on bone (31, 44, 45), while others have shown the exact opposite (46, 47).

This study has found the potential benefits of n-3 PUFA in the prevention or treatment of bone loss by increasing osteogenesis in depressed participants. Although the effects of n-3 PUFA supplementation on bone turnover markers and BMD have been investigated in osteopenia, pre- and post-menopausal, overweight, kidney transplant recipients, and healthy individuals (48–53), very few studies have evaluated those in depression. Only one study found no significant change in CTX levels in participants with mild-to-moderate depression after 12 weeks of supplementation with n-3 PUFAs (1.48 g/d) but without testing bone formation markers (54). Previous systematic reviews and meta-analyses have revealed an inverse association between n-3 PUFA and fracture risk, and n-3 PUFA may improve BMD, although the evidence was of low quality (55, 56). In addition, a recent meta-analysis found that n-3 PUFA had some potential benefits for younger postmenopausal subjects in the short term. However, it might not affect BMD or bone metabolism markers (57). Another meta-analysis showed that n-3 PUFA significantly affected bone turnover markers (58). This is the first study to show that high n-3 PUFA supplements could increase bone formation markers in younger first-diagnosed depressed participants (mean age 26.5 years).

The mechanisms underlying the benefits of n-3 PUFA are multi-factorial. Previous studies suggested that EPA and DHA supplementation could reduce the expression of multiple inflammatory markers involved in the pathogenesis of osteoporosis, such as CRP, IL-6, and TNF-α (59–63). Besides, EPA-derived Resolvin E1 (RvE1) has also been reported to reduce systemic inflammation and protect against inflammation-induced bone loss (64, 65). In addition, n-3 PUFA supplementation has prevented age-related bone and hematopoietic bone marrow (HBM) loss by reducing MAT expansion (66). Furthermore, *in vitro* studies supported that DHA has widened the whole skull width of mice by stimulating osteoblast activity (22). EPA and DHA can decrease the number of osteoclasts and impede osteoclast differentiation through the GRP120-related signaling pathway (67). However, previous findings have suggested different actions between EPA and DHA on bone cells. DHA strongly inhibited bone marrow-derived macrophages into osteoclasts *in vitro* by inhibiting sRANKL, while EPA enhanced it (68). The effect of EPA on osteoclastogenesis may be influenced by the different effects of its metabolite, such as resolvin E1 and PGE3 (69, 70). In summary, the osteoprotective effects of n-3 PUFA through various mechanisms of action are complex, which should be considered when employing nutritional supplements of n-3 PUFA as therapy for bone loss in depression in the future.

This study has shown that improving bone metabolism markers, which is a decrease in bone resorption and an increase in bone formation, predicted clinical symptomatic outcomes. Since only 40–60% of participants respond clinically, and only 30–45% achieve clinical remission after pharmaceutical treatment in depression (71, 72), it is crucial to predict treatment response. This is the first study to find bone turnover markers could predict treatment outcomes of anxiety symptoms in depression. Further research is needed on this topic.

This study has several limitations. The present study only measured peripheral bone turnover markers to assess changes in bone metabolism instead of the gold standard, such as dual-energy X-ray absorptiometry (DEXA) and quantitative computed tomography (QCT). Although a 12-week follow-up period is considered sufficient to assess changes in bone turnover in the short term (54), direct prediction of changes in long-term bone health in depressed participants from the current results may be insufficient. Therefore, future studies are needed to investigate the effects of long-term n-3 PUFA supplementation on bone turnover markers and BMD. Secondly, unlike animal studies, clinical studies need to consider many confounding factors such as disease, drug use, smoking, alcohol consumption, age, gender, dietary status, lifestyle habits, body mass index, etc., and the present study did not collect and analyze data on all these confounding factors. Thirdly, immune inflammation-related mechanisms are essential for depression-related bone loss and bone protection effect induced by n-3 PUFA, while due to the limited clinical samples, this study did not collect inflammatory factors. Fourthly, all participants have been prescribed venlafaxine by the recommendation from psychiatrists with a dosage range of 75–225 mg/day. However, neither the blood levels of the drug nor the related neurotransmitters were measured in the study. The sex difference of bone turnover markers (73) and is worth further investigating. However, in this study, due to the small sample size (*n* = 36) of the n-3 PUFA and placebo group, the analysis of sex differences was not conducted. In our study, the fish oil was a mixture. It is difficult to evaluate the efficacy of fish oil from EPA or DHA since there were no pure EPA and pure DHA intervention groups. Finally, an exploratory analysis was conducted to investigate the potential interaction between bone loss and clinical characteristics; more relevant research is needed to confirm this result.

In conclusion, this study has revealed that individuals experiencing first-diagnosed, drug-naïve depression display heightened levels of bone formation. Intriguingly, the use of venlafaxine demonstrates a decrease in the bone remodeling process. Furthermore, fish oil supplementation unveils a notable increase in bone formation. These findings shed valuable light on potential strategies for mitigating and addressing bone loss in depression, prompting further exploration into preventive and therapeutic interventions for this complex relationship.

## Data Availability

The raw data supporting the conclusions of this article will be made available by the authors, without undue reservation.

## References

[1] Michelson D Stratakis C Hill L Reynolds J Galliven E Chrousos G. Bone mineral density in women with depression. N Engl J Med. (1996) 335:1176–81. doi: 10.1056/NEJM199610173351602, PMID: 8815939

[2] Li C Palka JM Abdullah N Adler-Neal A Banner B Efseroff B. Link between depression and bone mineral density in Cooper Center longitudinal study: indirect effects of vitamin D, inflammation, and physical activity. J Affect Disord. (2024) 344:277–83. doi: 10.1016/j.jad.2023.10.062, PMID: 37827262

[3] Spangler L Scholes D Brunner RL Robbins J Reed SD Newton KM. Depressive symptoms, bone loss, and fractures in postmenopausal women. J Gen Intern Med. (2008) 23:567–74. doi: 10.1007/s11606-008-0525-0, PMID: 18286345 PMC2324136

[4] Yazici KM Akinci A Sütçü A Ozçakar L. Bone mineral density in premenopausal women with major depressive disorder. Psychiatry Res. (2003) 117:271–5. doi: 10.1016/S0165-1781(03)00017-9, PMID: 12686369

[5] Chen K Wang T Tong X Song Y Hong J Sun Y. Osteoporosis is associated with depression among older adults: a nationwide population-based study in the USA from 2005 to 2020. Public Health. (2024) 226:27–31. doi: 10.1016/j.puhe.2023.10.022, PMID: 37988825

[6] Compston JE McClung MR Leslie WD. Osteoporosis. Lancet. (2019) 393:364–76. doi: 10.1016/S0140-6736(18)32112-3, PMID: 30696576

[7] Williams LJ Pasco JA Jacka FN Henry MJ Dodd S Berk M. Depression and bone metabolism. A review. Psychother Psychosom. (2009) 78:16–25. doi: 10.1159/000162297 18852498

[8] Ortuño MJ Robinson ST Subramanyam P Paone R Huang Y-Y Guo XE. Serotonin-reuptake inhibitors act centrally to cause bone loss in mice by counteracting a local anti-resorptive effect. Nat Med. (2016) 22:1170–9. doi: 10.1038/nm.4166, PMID: 27595322 PMC5053870

[9] Rizzoli R Cooper C Reginster J-Y Abrahamsen B Adachi JD Brandi ML. Antidepressant medications and osteoporosis. Bone. (2012) 51:606–13. doi: 10.1016/j.bone.2012.05.018, PMID: 22659406

[10] Luo Y Kataoka Y Ostinelli EG Cipriani A Furukawa TA. National prescription patterns of antidepressants in the treatment of adults with major depression in the US between 1996 and 2015: a population representative survey based analysis. Front Psychol. (2020) 11:35. doi: 10.3389/fpsyt.2020.00035, PMID: 32116850 PMC7033625

[11] Bolo NR Hodé Y Macher J-P. Long-term sequestration of fluorinated compounds in tissues after fluvoxamine or fluoxetine treatment: a fluorine magnetic resonance spectroscopy study in vivo. MAGMA. (2004) 16:268–76. doi: 10.1007/s10334-004-0033-0, PMID: 15042463

[12] Bruun SB Petersen I Kristensen NR Cronin-Fenton D Pedersen AB. Selective serotonin reuptake inhibitor use in hip fracture patients: a Danish nationwide prevalence study. Acta Orthop. (2019) 90:33–9. doi: 10.1080/17453674.2018.1543842, PMID: 30526179 PMC6366466

[13] Yadav VK Ryu J-H Suda N Tanaka KF Gingrich JA Schütz G. Lrp5 controls bone formation by inhibiting serotonin synthesis in the duodenum. Cell. (2008) 135:825–37. doi: 10.1016/j.cell.2008.09.059, PMID: 19041748 PMC2614332

[14] Warden SJ Haney EM. Skeletal effects of serotonin (5-hydroxytryptamine) transporter inhibition: evidence from in vitro and animal-based studies. J Musculoskelet Neuronal Interact. (2008) 8:121–32. PMID: 18622081 PMC4155922

[15] Moura C Bernatsky S Abrahamowicz M Papaioannou A Bessette L Adachi J. Antidepressant use and 10-year incident fracture risk: the population-based Canadian multicentre osteoporosis study (CaMoS). Osteoporos Int. (2014) 25:1473–81. doi: 10.1007/s00198-014-2649-x, PMID: 24566587 PMC5094888

[16] Ferroni L Gardin C Bellin G Vindigni V Pavan C Zavan B. Effects of novel antidepressant drugs on mesenchymal stem cell physiology. Biomed Pharmacother. (2019) 114:108853. doi: 10.1016/j.biopha.2019.108853, PMID: 30986624

[17] Tanaka K Hirai T Kodama D Kondo H Hamamura K Togari A. α1B -Adrenoceptor signalling regulates bone formation through the up-regulation of CCAAT/enhancer-binding protein δ expression in osteoblasts. Br J Pharmacol. (2016) 173:1058–69. doi: 10.1111/bph.13418, PMID: 26750808 PMC5341235

[18] Zhu Y Ma Y Elefteriou F. Cortical bone is an extraneuronal site of norepinephrine uptake in adult mice. Bone Rep. (2018) 9:188–98. doi: 10.1016/j.bonr.2018.11.002, PMID: 30581894 PMC6296164

[19] Ma Y Krueger JJ Redmon SN Uppuganti S Nyman JS Hahn MK. Extracellular norepinephrine clearance by the norepinephrine transporter is required for skeletal homeostasis. J Biol Chem. (2013) 288:30105–13. doi: 10.1074/jbc.M113.481309, PMID: 24005671 PMC3798479

[20] Rizzoli R Reginster J-Y Boonen S Bréart G Diez-Perez A Felsenberg D. Adverse reactions and drug-drug interactions in the management of women with postmenopausal osteoporosis. Calcif Tissue Int. (2011) 89:91–104. doi: 10.1007/s00223-011-9499-8, PMID: 21637997 PMC3135835

[21] Tao SS Wang P Wang XY Yin KJ Yang XK Wang ZX. Causal effect of polyunsaturated fatty acids on bone mineral density and fracture. Front Nutr. (2022) 9:1014847. doi: 10.3389/fnut.2022.1014847, PMID: 36570136 PMC9772990

[22] Ahn SH Park S-Y Baek J-E Lee S-Y Baek W-Y Lee S-Y. Free fatty acid receptor 4 (GPR120) stimulates bone formation and suppresses bone resorption in the presence of elevated n-3 fatty acid levels. Endocrinology. (2016) 157:2621–35. doi: 10.1210/en.2015-1855, PMID: 27145004

[23] Lu A-X Lin Y Li J Liu J-X Yan C-H Zhang L. Effects of food-borne docosahexaenoic acid supplementation on bone lead mobilisation, mitochondrial function and serum metabolomics in pre-pregnancy lead-exposed lactating rats. Environ Pollut. (2023) 337:122613. doi: 10.1016/j.envpol.2023.122613, PMID: 37757928

[24] Haag M Magada ON Claassen N Böhmer LH Kruger MC. Omega-3 fatty acids modulate ATPases involved in duodenal ca absorption. Prostaglandins Leukot Essent Fat Acids. (2003) 68:423–9. doi: 10.1016/S0952-3278(03)00067-X, PMID: 12798663

[25] Kirkup SE Cheng Z Elmes M Wathes DC Abayasekara DRE. Polyunsaturated fatty acids modulate prostaglandin synthesis by ovine amnion cells in vitro. Reproduction. (2010) 140:943–51. doi: 10.1530/REP-09-0575, PMID: 20826537

[26] Zaloga GP. Narrative review of n-3 polyunsaturated fatty acid supplementation upon immune functions, resolution molecules and lipid peroxidation. Nutrients. (2021) 13:662. doi: 10.3390/nu13020662, PMID: 33670710 PMC7922327

[27] Wang L Liu T Guo J Zhao T Tang H Jin K. Abnormal erythrocyte fatty acid composition in first-diagnosed, drug-naïve patients with depression. J Affect Disord. (2022) 318:414–22. doi: 10.1016/j.jad.2022.09.023, PMID: 36113689

[28] Yang R Wang L Jin K Cao S Wu C Guo J. Omega-3 polyunsaturated fatty acids supplementation alleviate anxiety rather than depressive symptoms among first-diagnosed, drug-Naïve major depressive disorder patients: a randomized clinical trial. Front Nutr. (2022) 9:876152. doi: 10.3389/fnut.2022.876152, PMID: 35903448 PMC9315396

[29] Wippert P-M Block A Mansuy IM Peters EMJ Rose M Rapp MA. Alterations in bone homeostasis and microstructure related to depression and Allostatic load. Psychother Psychosom. (2019) 88:383–5. doi: 10.1159/000503640, PMID: 31639808

[30] Wuertz-Kozak K Roszkowski M Cambria E Block A Kuhn GA Abele T. Effects of early life stress on bone homeostasis in mice and humans. Int J Mol Sci. (2020) 21:6634. doi: 10.3390/ijms21186634, PMID: 32927845 PMC7556040

[31] Yirmiya R Goshen I Bajayo A Kreisel T Feldman S Tam J. Depression induces bone loss through stimulation of the sympathetic nervous system. Proc Natl Acad Sci USA. (2006) 103:16876–81. doi: 10.1073/pnas.0604234103, PMID: 17075068 PMC1636547

[32] Li S Qui Y Teng Z Chen J Kang D Tang H. Association between bipolar disorder and low bone mass: a cross-sectional study with newly diagnosed, drug-Naïve patients. Front Psychol. (2020) 11:530. doi: 10.3389/fpsyt.2020.00530 32587534 PMC7299052

[33] Li S Qiu Y Teng Z Xu B Tang H Xiang H. Research on biochemical indexes of bone metabolism in bipolar disorder: a cross-sectional study with newly diagnosed, drug-naïve patients. J Psychiatr Res. (2022) 151:197–204. doi: 10.1016/j.jpsychires.2022.04.015, PMID: 35500447

[34] Williams LJ Berk M Hodge JM Kotowicz MA Stuart AL Chandrasekaran V. Selective serotonin reuptake inhibitors (SSRIs) and markers of bone turnover in men. Calcif Tissue Int. (2018) 103:125–30. doi: 10.1007/s00223-018-0398-0, PMID: 29441424

[35] Rawson KS Dixon D Civitelli R Peterson TR Mulsant BH Reynolds CF. Bone turnover with venlafaxine treatment in older adults with depression. J Am Geriatr Soc. (2017) 65:2057–63. doi: 10.1111/jgs.14936, PMID: 28555718 PMC5626017

[36] Shea MLO Garfield LD Teitelbaum S Civitelli R Mulsant BH Reynolds CF. Serotonin-norepinephrine reuptake inhibitor therapy in late-life depression is associated with increased marker of bone resorption. Osteoporos Int. (2013) 24:1741–9. doi: 10.1007/s00198-012-2170-z, PMID: 23358607 PMC4066460

[37] Aydin H Mutlu N Akbas NBG. Treatment of a major depression episode suppresses markers of bone turnover in premenopausal women. J Psychiatr Res. (2011) 45:1316–20. doi: 10.1016/j.jpsychires.2011.04.005, PMID: 21531430

[38] Diem SJ Joffe H Larson JC Tsai JN Guthrie KA LaCroix AZ. Effects of escitalopram on markers of bone turnover: a randomized clinical trial. J Clin Endocrinol Metab. (2014) 99:E1732–7. doi: 10.1210/jc.2014-2288, PMID: 25014001 PMC4154080

[39] Bonnet N Bernard P Beaupied H Bizot JC Trovero F Courteix D. Various effects of antidepressant drugs on bone microarchitectecture, mechanical properties and bone remodeling. Toxicol Appl Pharmacol. (2007) 221:111–8. doi: 10.1016/j.taap.2007.02.005, PMID: 17383703

[40] Lee SH Mastronardi CA Li RW Paz-Filho G Dutcher EG Lewis MD. Short-term antidepressant treatment has long-lasting effects, and reverses stress-induced decreases in bone features in rats. Transl Psychiatry. (2019) 9:10. doi: 10.1038/s41398-018-0351-z, PMID: 30664741 PMC6341077

[41] Zhang H Li K Zhao Y Zhang Y Sun J Li S. Long-term use of fluoxetine accelerates bone loss through the disruption of sphingolipids metabolism in bone marrow adipose tissue. Transl Psychiatry. (2020) 10:138. doi: 10.1038/s41398-020-0819-5, PMID: 32398744 PMC7217841

[42] Gustafsson BI Thommesen L Stunes AK Tommeras K Westbroek I Waldum HL. Serotonin and fluoxetine modulate bone cell function in vitro. J Cell Biochem. (2006) 98:139–51. doi: 10.1002/jcb.20734, PMID: 16408289

[43] Battaglino R Fu J Späte U Ersoy U Joe M Sedaghat L. Serotonin regulates osteoclast differentiation through its transporter. J Bone Miner Res. (2004) 19:1420–31. doi: 10.1359/JBMR.040606, PMID: 15312242

[44] Battaglino R Vokes M Schulze-Späte U Sharma A Graves D Kohler T. Fluoxetine treatment increases trabecular bone formation in mice. J Cell Biochem. (2007) 100:1387–94. doi: 10.1002/jcb.21131, PMID: 17041947

[45] Lam RW Wong H-K Kumarsing RA Chua AN Ho RC McIntyre RS. Fluoxetine improves bone microarchitecture and mechanical properties in rodents undergoing chronic mild stress - an animal model of depression. Transl Psychiatry. (2022) 12:339. doi: 10.1038/s41398-022-02083-w, PMID: 35987907 PMC9392792

[46] Warden SJ Nelson IR Fuchs RK Bliziotes MM Turner CH. Serotonin (5-hydroxytryptamine) transporter inhibition causes bone loss in adult mice independently of estrogen deficiency. Menopause. (2008) 15:1176–83. doi: 10.1097/gme.0b013e318173566b, PMID: 18725867

[47] Warden SJ Robling AG Sanders MS Bliziotes MM Turner CH. Inhibition of the serotonin (5-hydroxytryptamine) transporter reduces bone accrual during growth. Endocrinology. (2005) 146:685–93. doi: 10.1210/en.2004-1259, PMID: 15539550

[48] Fonolla-Joya J Reyes-García R García-Martín A López-Huertas E Muñoz-Torres M. Daily intake of Milk enriched with n-3 fatty acids, oleic acid, and calcium improves metabolic and bone biomarkers in postmenopausal women. J Am Coll Nutr. (2016) 35:529–36. doi: 10.1080/07315724.2014.1003114, PMID: 27463412

[49] Lappe J Kunz I Bendik I Prudence K Weber P Recker R. Effect of a combination of genistein, polyunsaturated fatty acids and vitamins D3 and K1 on bone mineral density in postmenopausal women: a randomized, placebo-controlled, double-blind pilot study. Eur J Nutr. (2013) 52:203–15. doi: 10.1007/s00394-012-0304-x, PMID: 22302614 PMC3549413

[50] LeBoff MS Chou SH Murata EM Donlon CM Cook NR Mora S. Effects of supplemental vitamin D on bone health outcomes in women and men in the VITamin D and OmegA-3 TriaL (VITAL). J Bone Miner Res. (2020) 35:883–93. doi: 10.1002/jbmr.3958, PMID: 31923341 PMC7217747

[51] Lucey AJ Paschos GK Cashman KD Martínéz JA Thorsdottir I Kiely M. Influence of moderate energy restriction and seafood consumption on bone turnover in overweight young adults. Am J Clin Nutr. (2008) 87:1045–52. doi: 10.1093/ajcn/87.4.1045, PMID: 18400730

[52] Tartibian B Hajizadeh Maleki B Kanaley J Sadeghi K. Long-term aerobic exercise and omega-3 supplementation modulate osteoporosis through inflammatory mechanisms in post-menopausal women: a randomized, repeated measures study. Nutr Metab (Lond). (2011) 8:71. doi: 10.1186/1743-7075-8-71, PMID: 21999620 PMC3212907

[53] Vanlint SJ Ried K. Efficacy and tolerability of calcium, vitamin D and a plant-based omega-3 oil for osteopenia: a pilot RCT. Maturitas. (2012) 71:44–8. doi: 10.1016/j.maturitas.2011.10.004, PMID: 22078660

[54] Appleton KM Fraser WD Rogers PJ Ness AR Tobias JH. Supplementation with a low-moderate dose of n-3 long-chain PUFA has no short-term effect on bone resorption in human adults. Br J Nutr. (2011) 105:1145–9. doi: 10.1017/S0007114510004861, PMID: 21129235

[55] the PUFAH Group Abdelhamid A Hooper L Sivakaran R Hayhoe RPG Welch A. The relationship between Omega-3, Omega-6 and Total polyunsaturated fat and musculoskeletal health and functional status in adults: a systematic review and Meta-analysis of RCTs. Calcif Tissue Int. (2019) 105:353–72. doi: 10.1007/s00223-019-00584-3, PMID: 31346665

[56] Sadeghi O Djafarian K Ghorabi S Khodadost M Nasiri M Shab-Bidar S. Dietary intake of fish, n-3 polyunsaturated fatty acids and risk of hip fracture: a systematic review and meta-analysis on observational studies. Crit Rev Food Sci Nutr. (2019) 59:1320–33. doi: 10.1080/10408398.2017.1405908, PMID: 29244536

[57] Gao J Xie C Yang J Tian C Zhang M Lu Z. The effects of n-3 PUFA supplementation on bone metabolism markers and body bone mineral density in adults: a systematic review and Meta-analysis of RCTs. Nutrients. (2023) 15:2806. doi: 10.3390/nu15122806, PMID: 37375709 PMC10303698

[58] Dou Y Wang Y Chen Z Yu X Ma D. Effect of n-3 polyunsaturated fatty acid on bone health: a systematic review and meta-analysis of randomized controlled trials. Food Sci Nutr. (2022) 10:145–54. doi: 10.1002/fsn3.2655, PMID: 35035917 PMC8751426

[59] Hajishengallis G Chavakis T. Local and systemic mechanisms linking periodontal disease and inflammatory comorbidities. Nat Rev Immunol. (2021) 21:426–40. doi: 10.1038/s41577-020-00488-6, PMID: 33510490 PMC7841384

[60] KubotaYHigashiyamaAImanoHSugiyamaDKawamuraKKadotaA. Serum polyunsaturated fatty acid composition and serum high-sensitivity C-reactive protein levels in healthy Japanese residents: the KOBE study. J Nutr Health Aging. (2015) 19:719–28. doi: 10.1007/s12603-015-0497-9, PMID: 26193854

[61] Li K Huang T Zheng J Wu K Li D. Effect of marine-derived n-3 polyunsaturated fatty acids on C-reactive protein, interleukin 6 and tumor necrosis factor α: a meta-analysis. PLoS One. (2014) 9:e88103. doi: 10.1371/journal.pone.0088103, PMID: 24505395 PMC3914936

[62] Place DE Malireddi RKS Kim J Vogel P Yamamoto M Kanneganti T-D. Osteoclast fusion and bone loss are restricted by interferon inducible guanylate binding proteins. Nat Commun. (2021) 12:496. doi: 10.1038/s41467-020-20807-8, PMID: 33479228 PMC7820603

[63] Taha AM Shaarawy AS Omar MM Abouelmagd K Shalma NM Alhashemi M. Effect of Omega-3 fatty acids supplementation on serum level of C-reactive protein in patients with COVID-19: a systematic review and meta-analysis of randomized controlled trials. J Transl Med. (2022) 20:401. doi: 10.1186/s12967-022-03604-3, PMID: 36064554 PMC9444081

[64] Hasturk H Kantarci A Goguet-Surmenian E Blackwood A Andry C Serhan CN. Resolvin E1 regulates inflammation at the cellular and tissue level and restores tissue homeostasis in vivo. J Immunol. (2007) 179:7021–9. doi: 10.4049/jimmunol.179.10.7021, PMID: 17982093

[65] Hasturk H Kantarci A Ohira T Arita M Ebrahimi N Chiang N. RvE1 protects from local inflammation and osteoclast- mediated bone destruction in periodontitis. FASEB J. (2006) 20:401–3. doi: 10.1096/fj.05-4724fje, PMID: 16373400

[66] Bani Hassan E Alderghaffar M Wauquier F Coxam V Demontiero O Vogrin S. The effects of dietary fatty acids on bone, hematopoietic marrow and marrow adipose tissue in a murine model of senile osteoporosis. Aging (Albany NY). (2019) 11:7938–47. doi: 10.18632/aging.102299, PMID: 31553309 PMC6781972

[67] Kasonga AE Kruger MC Coetzee M. Free fatty acid receptor 4-β-arrestin 2 pathway mediates the effects of different classes of unsaturated fatty acids in osteoclasts and osteoblasts. Biochim Biophys Acta Mol Cell Biol Lipids. (2019) 1864:281–9. doi: 10.1016/j.bbalip.2018.12.009, PMID: 30578965

[68] Benova A Ferencakova M Bardova K Funda J Prochazka J Spoutil F. Omega-3 PUFAs prevent bone impairment and bone marrow adiposity in mouse model of obesity. Commun Biol. (2023) 6:1043. doi: 10.1038/s42003-023-05407-8, PMID: 37833362 PMC10575870

[69] Zhu M Van Dyke TE Gyurko R. Resolvin E1 regulates osteoclast fusion via DC-STAMP and NFATc1. FASEB J. (2013) 27:3344–53. doi: 10.1096/fj.12-220228, PMID: 23629863 PMC3714580

[70] Akiyama M Nakahama K-i Morita I. Impact of docosahexaenoic acid on gene expression during osteoclastogenesis in vitro--a comprehensive analysis. Nutrients. (2013) 5:3151–62. doi: 10.3390/nu5083151, PMID: 23945674 PMC3775247

[71] Carvalho AF Cavalcante JL Castelo MS Lima MCO. Augmentation strategies for treatment-resistant depression: a literature review. J Clin Pharm Ther. (2007) 32:415–28. doi: 10.1111/j.1365-2710.2007.00846.x, PMID: 17875106

[72] Khin NA Chen Y-F Yang Y Yang P Laughren TP. Exploratory analyses of efficacy data from major depressive disorder trials submitted to the US Food and Drug Administration in support of new drug applications. J Clin Psychiatry. (2011) 72:464–72. doi: 10.4088/JCP.10m06191, PMID: 21527123

[73] Choi KH Lee JH Lee DG. Sex-related differences in bone metabolism in osteoporosis observational study. Medicine. (2021) 100:e26153. doi: 10.1097/MD.0000000000038769, PMID: 34032772 PMC8154389

